# Floating septum technique: easy and safe method maxillary sinus septa in sinus lifting procedure

**DOI:** 10.1186/s40902-019-0233-1

**Published:** 2019-11-28

**Authors:** Junho Jung, Bo-Yeon Hwang, Byung-Soo Kim, Jung-Woo Lee

**Affiliations:** 1 Department of Oral and Maxillofacial Surgery, School of Dentistry, Kyung Hee University, 26, Kyungheedae-ro, Dongdaemun-gu, 02447 Seoul, Republic of Korea; 2 Department of Oral and Maxillofacial Surgery, Kyung Hee University Dental Hospital, Seoul, Republic of Korea

**Keywords:** Maxillary sinus, Sinus elevation, Septum

## Abstract

**Background:**

The presence of septa increases the risk of Schneiderian membrane perforation during sinus lift procedure, and therefore, the chance of graft failure increases. We present a safe method of managing septa and, in particular, overcoming small and palatally located septa.

**Methods:**

After the elevation of the flap and the creation of a small bony window positioned anterior to the septum, the Schneiderian membrane is lifted carefully. A thin and narrow osteotome is then placed at the indentation created at the base of the septum, and mobilization of the septum is achieved by gentle malleting. The membrane is again carefully lifted up behind the septum.

**Results:**

There was one small membrane perforation case in all 16 cases, and none of these patients showed postoperative complications such as implant failure, infection, or maxillary sinusitis.

**Conclusions:**

This technique is useful for overcoming the problem of maxillary sinus septa hindering the sinus floor elevation procedure, leading to fewer complications.

## Background

Sinus lift is a popular method for the placement of dental implants in the atrophic posterior maxillary area. Various causes of Schneiderian membrane perforation have been reported [1], and the presence of septa increases the risk of Schneiderian membrane perforation during this procedure, and therefore, the chance of graft failure increases. The prevalence of maxillary septa is reported to be 7.1–58.3% [2], which is too high to ignore. As expected, the frequency of perforation is higher in the presence of septa (42.9%) than in the absence of septa (23.8%) [3]. Various approaches to overcome the increased risk of perforation in the presence of septa have been proposed [4, 5]. However, these methods involve making wider windows to elevate the membrane around the septa and cannot be used on the small septa located in the palatal area. For these reasons, we will describe here a safe method of managing septa and, in particular, overcoming small septa.

## Methods

### Surgical technique

A crestal incision is made with a vertical releasing incision on the mesial and distal sides. The flap is then subperiosteally elevated. It is sufficient to create a single small window. The distal margin of the bony window is positioned anterior to the septum or extended distally to include the septum. The size of the bony window should be large enough to allow access of the sinus lifting instruments used to elevate the membrane.

**Fig 1:**
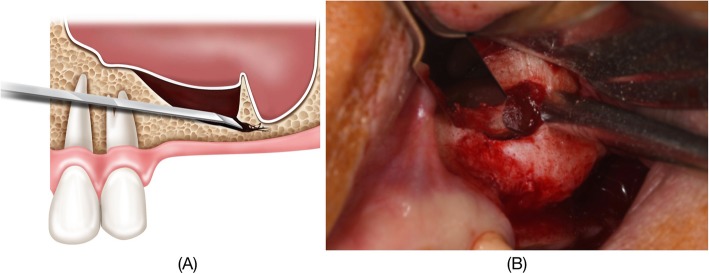
**a** A schematic image of the floating septum technique. **b** A clinical photograph showing the application of a thin and narrow osteotome after the creation of bony window and the elevation of Schneiderian membrane

Subsequently, the Schneiderian membrane is lifted carefully on all sides except at the septum. After this preparation is complete, a linear indentation with a round bur or piezoelectric instrument is made at the base of the septum. A thin and narrow osteotome is then placed at the indentation (Fig. 1). To avoid tearing the membrane, osteotomy should be performed with gentle malleting. Mobilization of the septum is achieved and the membrane is again carefully lifted up behind the septum. The septum is now floating along with the membrane. Removal of the septum is not recommended, as this can cause an undesirable perforation. The Schneiderian membrane should then be macroscopically inspected and the graft material inserted.

## Results

We have operated on 16 patients (16 sides) (Table 1). There were eleven men and five women, mean (SD) age was 53.63 (8.94) years. All patients received the installation of dental implants and sinus lifting procedure simultaneously. Only one patient had a small Schneiderian membrane perforation which was repaired with Bio-Gide® (Geistlich Pharma AG®, Wolhusen, Switzerland), and none of these patients showed postoperative complications such as implant failure, infection, or maxillary sinusitis.

**Table 1:** Patients’ details

Case no.	Age (years)	Sex	Dental implants	Surgery	Septum management	Complications
1	58	M	#14, 15, 16, 17	SL + DI + GBR	O	No
2	63	F	#26	SL + DI	O	No
3	68	M	#26, 27	SL + DI	O	No
4	46	M	#26	TE + SL + IDI	O	No
5	64	M	#15, 16, 17	SL + DI	O	No
6	42	M	#16	TE + SL + IDI	O	No
7	46	F	#26, 27	SL + DI	O	No
8	46	M	#26, 27	SL + DI	O	No
9	64	M	#16, 17	SL + DI	O	Membrane perforation
10	56	F	#15, 16, 17	SL + DI + GBR	R + O	No
11	48	M	#26, 27	SL + DI	R + O	No
12	65	F	#26, 27	SL + DI	R + O	No
13	45	F	#16	TE + SL + IDI	P + O	No
14	55	M	#26, 27	SL + DI	P + O	No
15	45	M	#26, 26	SL + DI	O	No
16	47	M	#26, 27	SL + DI	P + O	No

*M* male, *F* female, *SL* sinus lift, *DI* dental implants, *GBR* guided bone regeneration, *TE* tooth extraction, *IDI* immediate dental implant installation, *O* osteotom, *R* round bur, *P* piezoelectric instrument

## Discussion

We obtained a favorable result with the method described herein, although further studies with larger sample sizes are required to validate our result. This technique only requires one small window, which means shorter operation times, decreased surgical discomfort, and better bony in-growth to the grafted area. In addition, since this method does not involve crossing the instrument tip of the septum, the risk of tearing the membrane is also decreased. This method was useful for managing the septum in the maxillary second molar region and immediate implant placement after maxillary molar extraction, which were quite challenging with the conventional method.

## Conclusion

We postulate that this technique is useful for overcoming the problem of maxillary sinus septa hindering the sinus floor elevation procedure, leading to fewer complications.

## Data Availability

Not applicable
